# Pyrroloquinoline Quinone (PQQ) Attenuates Hydrogen Peroxide-Induced Injury Through the Enhancement of Mitochondrial Function in Human Trabecular Meshwork Cells

**DOI:** 10.3390/ijms26146938

**Published:** 2025-07-19

**Authors:** Sabrina Petricca, Antonio Matrone, Daria Capece, Irene Flati, Vincenzo Flati, Enrico Ricevuto, Giuseppe Celenza, Nicola Franceschini, Mirco Mastrangelo, Cristina Pellegrini, Loredana Cristiano, Giuseppe Familiari, Benedetta Cinque, Giovanna Di Emidio, Carla Tatone, Roberto Iorio

**Affiliations:** 1 Department of Biotechnological and Applied Clinical Sciences, University of L’Aquila, 67100 L’Aquila, Italy; sabrina.petricca@univaq.it (S.P.); antonio.matrone@graduate.univaq.it (A.M.); daria.capece@univaq.it (D.C.); irene.flati@graduate.univaq.it (I.F.); vincenzo.flati@univaq.it (V.F.); enrico.ricevuto@univaq.it (E.R.); giuseppe.celenza@univaq.it (G.C.); nicola.franceschini@univaq.it (N.F.); mirco.mastrangelo@univaq.it (M.M.); cristina.pellegrini@univaq.it (C.P.); 2 Department of Anatomy, Histology, Forensic Medicine and Orthopaedics, Sapienza University, 00165 Rome, Italy; loredana.cristiano@uniroma1.it (L.C.); giuseppe.familiari@uniroma1.it (G.F.); 3 Department of Life, Health and Environmental Sciences, University of L’Aquila, 67100 L’Aquila, Italy; benedetta.cinque@univaq.it (B.C.); giovanna.diemidio@univaq.it (G.D.E.); carla.tatone@univaq.it (C.T.)

**Keywords:** human trabecular meshwork cells, pyrroloquinoline quinone (PQQ), mitochondrial bioenergetic, mitochondrial network morphology, SIRT1/PGC1-α pathway, SIRT3 signalling

## Abstract

Mitochondrial metabolism in the trabecular meshwork (TM) plays a critical role in maintaining intraocular pressure homeostasis by supporting the energy-demanding processes involved in aqueous humour outflow. In primary open-angle glaucoma, oxidative stress impairs mitochondrial function, leading to TM dysfunction. Therefore, understanding and targeting mitochondrial health in TM cells could offer a novel therapeutic strategy. Pyrroloquinoline quinone (PQQ) is a redox cofactor with antioxidant and mitochondrial-enhancing properties. However, its effects on human TM (HTM) cells remain largely unexplored. This study examined PQQ cytoprotective effects against H_2_O_2_-induced oxidative stress in HTM cells. Seahorse analyses revealed that PQQ alone improves mitochondrial respiration and ATP production. Moreover, PQQ mitigates H_2_O_2_-induced cellular damage and preserves mitochondrial function by normalising proton leak and increasing ATP levels. Furthermore, TEM and confocal microscopy showed that PQQ can partially alleviate structural damage, restoring mitochondrial network morphology, thereby leading to reduced cell death. Although these protective effects seem not to be mediated by changes in mitochondrial content or activation of the SIRT1/PGC1-α pathway, they may involve modulation of SIRT3, a key factor of mitochondrial metabolism and homeostasis. Overall, these results suggest that PQQ may represent a promising candidate for restoring mitochondrial function and reversing oxidative damage in HTM cells.

## 1. Introduction

The trabecular meshwork (TM) plays a crucial role in regulating aqueous humour outflow and maintaining intraocular pressure (IOP) homeostasis. Due to their high metabolic activity, TM cells demand an efficient energy balance and tight regulation of the cellular energy charge. This requires the presence of healthy mitochondria to ensure a constant energy supply for supporting critical processes, including extracellular matrix remodelling, cytoskeletal dynamics, and stress responses [1,2]. Given their dynamic and multifunctional nature, mitochondria perform several interconnected functions by exchanging complex signals between cellular compartments, cells, and even across organ systems [3,4,5,6,7]. They act as central hubs for sensing a wide array of cellular stressors and serve as crucial regulators of cell fate, orchestrating pathways that determine survival or programmed cell death [8,9,10]. To meet diverse cellular metabolic and functional demands, the mitochondrial network undergoes continuous remodelling through a set of tightly regulated processes, such as fission and fusion events, mitophagy, and mitochondrial biogenesis, all of which coordinate the organelle shape, size, and distribution. Under stress conditions, however, excessive production of reactive oxygen species (ROS) can damage mitochondrial DNA, impair oxidative phosphorylation, and disrupt mitochondrial dynamics [10,11,12]. Although multiple factors contribute to TM dysfunction, mitochondrial impairment and oxidative stress are key drivers, creating a damaging cycle that promotes compromised cellular bioenergetics, cellular senescence, chronic inflammation, apoptosis, and, ultimately, cell death [13,14,15,16,17,18,19]. These alterations not only lead to elevated IOP but may also adversely affect retinal ganglion cell health through the release of pro-inflammatory cytokines [20]. Therefore, preserving mitochondrial integrity in TM cells represents a critical and complementary strategy to conventional IOP-lowering treatments, particularly in patients for whom IOP control alone is insufficient, for preventing or mitigating glaucomatous neurodegeneration.

In the past few years, much attention has been paid to natural bioactive compounds with potential mitochondrial protective activity that can enhance mitochondrial function, counteract oxidative stress, and promote cellular resilience [21,22]. In this regard, quinones, a class of organic compounds, including pyrroloquinoline quinone (PQQ), have emerged as critical modulators of important biological activities [23,24].

PQQ is a natural compound found in various foods and commercially available as a dietary supplement in its disodium salt crystalline form. In 2018, the European Commission approved PQQ (marketed as MGCPQQ^®^) as a novel food ingredient, following a favourable safety assessment by the European Food Safety Authority (EFSA) [25]. In both humans and animal models, its concentrations in biological fluids and tissues typically range from 3 to 54 nM, depending on dietary intake, tissue type, and physiological conditions [26,27,28].

PQQ is a redox-active compound with potent antioxidant properties, known to play a protective role against mitochondrial dysfunction. Initially identified as a bacterial cofactor, PQQ has attracted attention in different in vitro and in vivo mammalian systems for its ability to modulate cellular energy metabolism and protect against oxidative stress. PQQ is an effective scavenger of superoxide and hydroxyl radicals [29], major contributors to mitochondrial dysfunction, and has been shown to counteract various forms of oxidative-stress-induced cellular damage, such as reoxygenation cardiac injury [30], murine hepatitis virus strain 3 (MHV-3)-induced liver injury [31], chronic heart failure [32], and hydrogen peroxide (H_2_O_2_)-induced apoptotic effects in nucleus pulposus cells [33]. Previous studies have also shown the ability of PQQ to promote mitochondrial biogenesis, modulate key regulators of mitochondrial metabolism, such as the peroxisome-proliferator-activated receptor gamma coactivator 1-alpha (PGC1-α) and sirtuins (SIRTs), and protect against mitochondrial dysfunction in various cell types and in vivo models [34,35,36,37,38]. One pivotal study demonstrated that PQQ stimulates mitochondrial biogenesis by activating the cyclic adenosine monophosphate (cAMP) response element-binding protein (CREB) and increasing PGC1-α expression in mouse hepatocytes [39]. More recently, PQQ has been shown to preserve mitochondrial function in auditory cells under oxidative stress by restoring the SIRT1/PGC1-α regulatory pathway, thereby enhancing mitochondrial biogenesis and overall mitochondrial performance [37]. Furthermore, PQQ also protects renal tubular epithelial cells from high glucose-induced oxidative stress and apoptosis by activating the phosphoinositide 3-kinase/protein kinase B/forkhead box O3a (PI3K/Akt/FoxO3a) signalling pathway and modulating SIRT3 expression [40].

Unlike these models, human TM (HTM) cells are uniquely subjected to both oxidative and mechanical stress due to their location within the aqueous outflow pathway and their constant exposure to fluctuating IOP. This distinctive combination of biomechanical and metabolic burdens underscores the need to investigate targeted mitochondrial-protective strategies in this cell type. Despite promising findings reported for PQQ in other tissues, evidence supporting its role in ocular health, especially in the anterior segment and TM cells, remains limited. Recently, Canovai et al. [41] demonstrated a neuroprotective effect of PQQ in in vitro models of retinal ganglion cell (RGC) degeneration, and Rossi et al. (2025) [42] reported functional improvements in RGCs in glaucoma patients following oral PQQ intake, as measured by pattern electroretinogram (PERG). Although these studies validate the systemic and retinal effects of PQQ, they do not address the anterior segment or the trabecular outflow pathway, the initial site of pathology in primary open-angle glaucoma.

In this study, we investigated the cytoprotective effects of PQQ against H_2_O_2_-induced oxidative and mitochondrial stress in HTM cells. We evaluated its impact on mitochondrial function and metabolic activity, structural integrity, and cell survival, aiming to uncover the molecular mechanisms through which PQQ may support TM cell health. To this end, the potential involvement of the SIRT1/PGC1-α signalling pathway and the modulation of SIRT3 were also assessed.

## 2. Results

### 2.1. Effects of PQQ on Cell Growth and Viability, Redox Homeostasis, and Mitochondrial Membrane Potential (ΔΨm) in Human Trabecular Meshwork Cells

**Figure 1:**
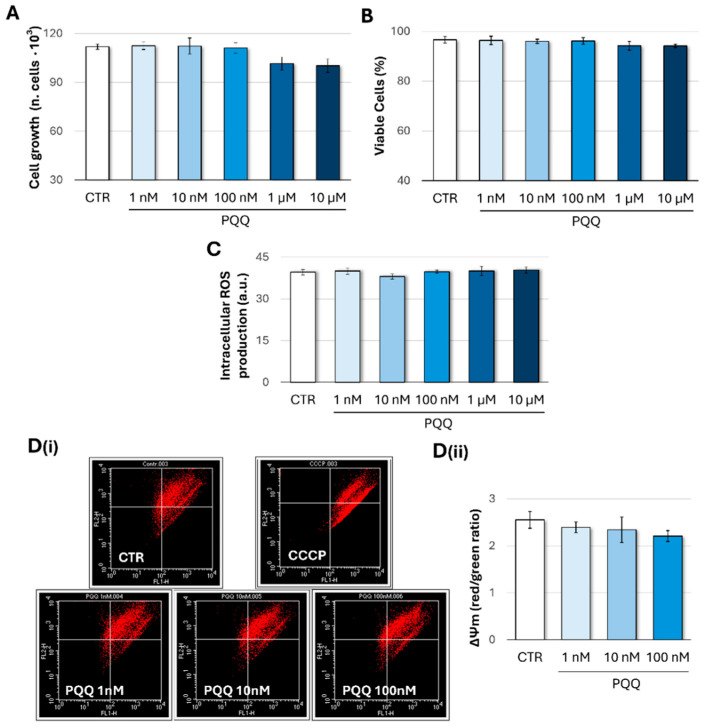
**PQQ does not alter cellular homeostasis, redox balance, and mitochondrial membrane potential (ΔΨm) in HTM cells**. HTM cells were exposed or not exposed (CTR) to increasing concentrations of PQQ for 24 h. Cell growth rate (**A**) and viability (**B**) were assessed using the trypan blue exclusion assay. (**C**) Intracellular ROS levels (a.u., arbitrary units) were measured using the fluorescent probe DCFH2-DA. (**D**(**i**)) Representative images of ΔΨm profiles obtained through staining with the potentiometric probe JC-1 and analysed by flow cytometry. (**D**(**ii**)) The graph shows the ΔΨm, expressed as the mean of the red/green fluorescence ratio of the mean fluorescence intensity, for each different experimental group. CCCP was used as a positive control for the abolishment of ΔΨm. Data from three independent experiments are presented as the mean ± SE; one-way ANOVA.

To assess the potential adverse effects of PQQ, HTM cell samples were exposed to increasing concentrations of quinone (1 nM–10 µM) for 24 h. Cell growth, viability, and intracellular reactive oxygen species (ROS) production were then evaluated. As shown in Figure 1A,B, no significant changes in proliferation or viability were observed, indicating the absence of cytotoxic effects. Likewise, ROS levels remained unchanged under the same conditions, suggesting that redox homeostasis was maintained (Figure 1C).

Given that trace amounts of PQQ (in a concentration range from picomolar to nanomolar) have been detected in human tissues, mitochondrial function was further evaluated using PQQ concentrations ranging from 1 to 100 nM. Mitochondrial membrane potential (ΔΨm) was assessed by using the JC-1 fluorescence dye, thus measuring the proportion of cells with high (orange-red fluorescence) or low (green fluorescence) ΔΨm. As shown in Figure 1D(ii), PQQ-treated cells exhibited a slight, non-significant decrease in mitochondrial polarisation (expressed as the red/green ratio) compared with controls, indicating that mitochondrial function was largely preserved.

Based on these findings, PQQ concentrations in the range of 1 nM to 100 nM were selected for subsequent experiments.

### 2.2. PQQ Increases Respiratory Capacity and ATP Production in HTM Cells

**Figure 2:**
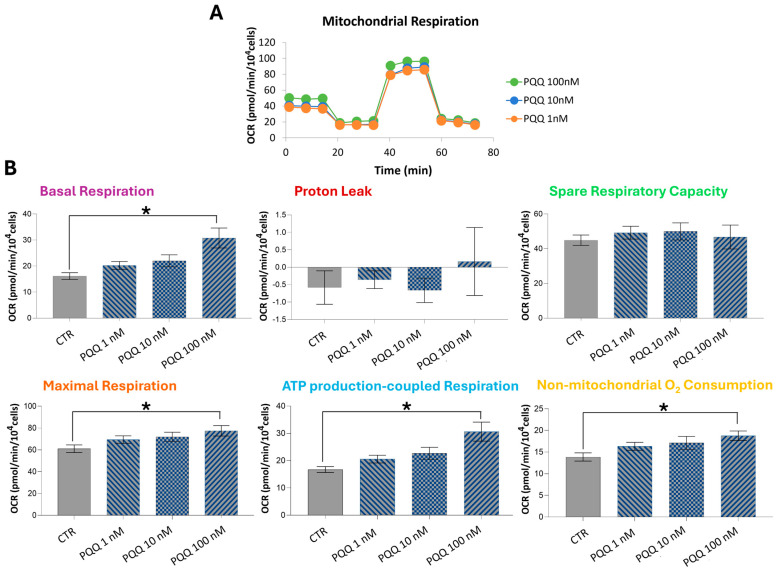
**PQQ enhances respiratory capacity and ATP production in HTM cells.** HTM cells were exposed to increasing concentrations of PQQ (1–100 nM) for 24 h. Bioenergetic parameters were analysed using the Seahorse XF flux analyser. (**A**) The graph shows the time-course profiles of oxygen consumption rate (OCR) in samples treated with different concentrations of PQQ. (**B**) The bar graphs represent the quantification of the following key parameters: “Basal respiration”, “Proton leak”, “Spare respiratory capacity”, “Maximal respiration”, “ATP production-coupled respiration”, and “Non-mitochondrial oxygen consumption”. Data from three independent experiments are expressed as the mean ± SE. One-way ANOVA followed by the Holm–Sidak post hoc test (* *p* < 0.05).

Although measurements of ΔΨm provide insights into the bioenergetic and functional status of mitochondria, they are neither sufficiently sensitive nor comprehensive for assessing oxidative phosphorylation activity and oxygen consumption rates (OCRs) [43]. To gain a deeper understanding of the effects of PQQ on the metabolic function of HTM cells, we analysed mitochondrial respiration by monitoring real-time changes in OCRs using Seahorse XF analysis (Figure 2A).

The resulting bioenergetic profiles suggest that PQQ induces significant remodelling of mitochondrial metabolic activity. Specifically, PQQ treatment led to a dose-dependent enhancement in key mitochondrial parameters, including basal respiration, maximal respiration, ATP-production-coupled respiration, and non-mitochondrial oxygen consumption (Figure 2B). However, these changes reached statistical significance only at the 100 nM PQQ concentration. Compared with the control group, cells treated with 100 nM PQQ exhibited a 1.9-fold increase in basal respiration, a 1.3-fold increase in maximal respiration, a 1.8-fold increase in ATP-linked respiration, and a 1.3-fold increase in non-mitochondrial oxygen consumption.

### 2.3. Short-Term Exposure to H_2_O_2_ Induces Concentration-Dependent Inhibitory Effects on Cell Viability and Alters the Mitochondrial Bioenergetics of HTM Cells

**Figure 3:**
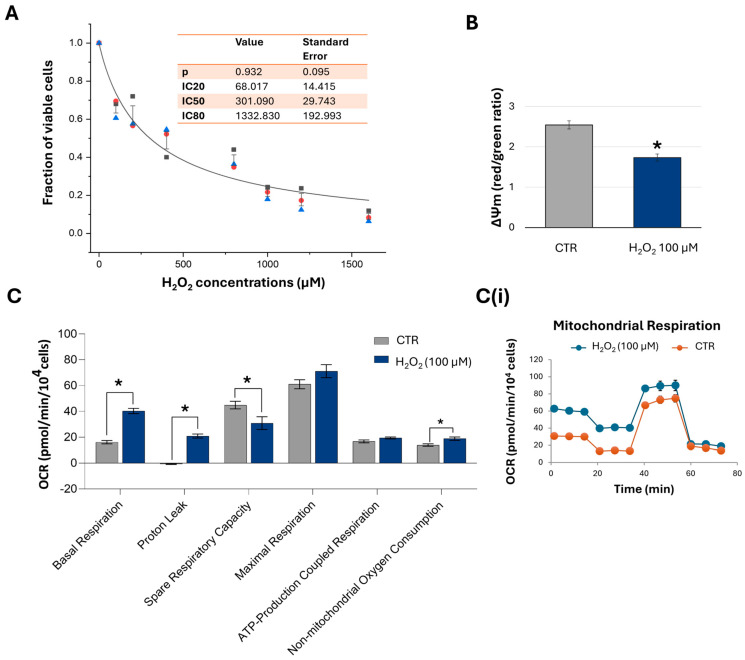
**Effects of hydrogen peroxide-induced stress on cell growth, viability, and mitochondrial function and bioenergetics in HTM cells.** HTM cells were exposed to a range of H_2_O_2_ concentrations (100–1600 µM) for 1 h. The dose–response curve (**A**) shows IC20, IC50, and IC80 values evaluated 24 h after exposure. Data from three independent experiments are presented as the mean ± SE, analysed by OriginPro software version 8.5 (OriginLab Corporation, Northampton, MA, USA). (**B**) Graph displays ΔΨm levels, expressed as the mean of the red/green fluorescence intensity ratio in control and H_2_O_2_ -treated (100 µM, for 1 h) cell populations. Data are expressed as mean ± SE from three independent experiments. * *p* < 0.05 by unpaired *t*-test. ((**C**,**C**(**i**))) Mitochondrial bioenergetic profiling of control and H_2_O_2_ -treated cells. Data are shown as mean ± SE from three independent experiments and multiple paired *t*-tests (* *p* < 0.05).

H_2_O_2_ is commonly used as a classical inducer of oxidative stress in in vitro models due to its ability to penetrate cell membranes and generate ROS. To establish an oxidative stress model, HTM cells were exposed to varying concentrations of H_2_O_2_ (100–1600 µM) for 1 h, followed by analysis of cell proliferation and viability 24 h post-treatment. Based on the resulting dose–response curve (Figure 3A), the inhibitory concentrations were determined as follows: IC_20_ = 68.02 µM ± 14.42; IC_50_ = 301.09 µM ± 29.74; and IC_80_ = 1332.83 µM ± 192.99. For subsequent experiments, the concentration of 100 µM H_2_O_2_ was selected to induce moderate oxidative stress and evaluate mitochondrial function and bioenergetic parameters. As shown in Figure 3B, H_2_O_2_ exposure led to a marked depolarisation of the ΔΨm, as evidenced by a greater than 40% reduction in the red/green fluorescence ratio. This loss of ΔΨm was accompanied by increases in basal respiration, proton leak, and non-mitochondrial oxygen consumption, along with a substantial decrease in spare respiratory capacity, indicating significant mitochondrial damage. Furthermore, these alterations occurred without a corresponding increase in ATP production, further supporting the presence of mitochondrial dysfunction (Figure 3C).

### 2.4. Protective Effects of PQQ Against H_2_O_2_-Induced Decline in Mitochondrial Respiratory Capacity in HTM Cells

**Figure 4:**
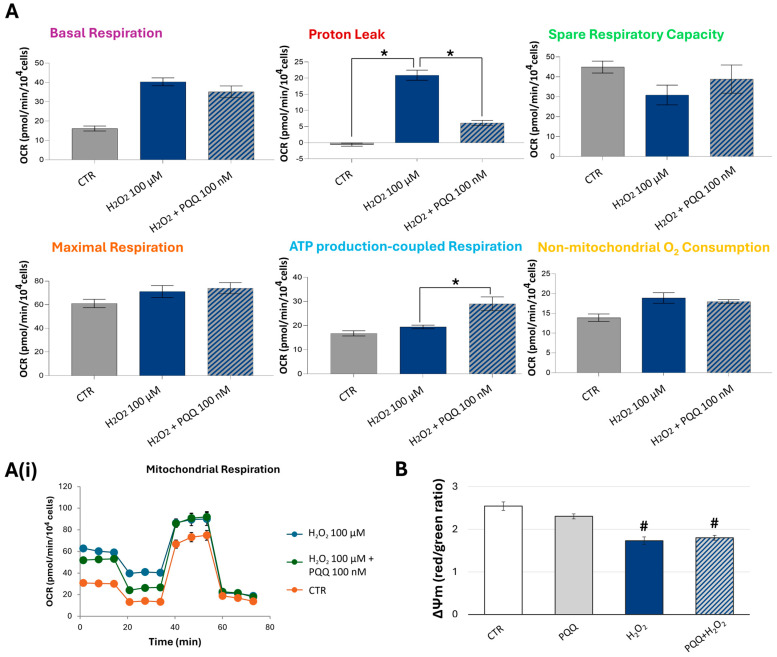
**Protective effects of PQQ against hydrogen peroxide-induced decline in mitochondrial respiratory capacity in HTM cells.** Following a 23 h pretreatment with PQQ and subsequent 1 h co-exposure to H_2_O_2_ (100 µM), all experimental groups were subjected to analysis of mitochondrial respiratory capacity (**A**) using the Seahorse analyser and mitochondrial membrane potential (ΔΨm) (**B**) by flow cytometry. (**A**) Bar graphs show the mean ± SE of the measured metabolic parameters. (**A**(**i**)) Time-course profiles of OCR in the different treated samples. (**B**) ΔΨm is expressed as the mean of the red/green fluorescence intensity ratio of mean fluorescence intensity (MFI). One-way ANOVA followed by the Holm–Sidak post hoc test (* *p* < 0.05; # *p* < 0.05 vs. CTR).

Given that 100 nM PQQ effectively induces mitochondrial bioenergetic remodelling, we next investigated whether exposure to this quinone could also confer protection against H_2_O_2_-induced mitochondrial damage. Following a 23 h pretreatment with PQQ, cells were co-exposed to 100 µM H_2_O_2_ for 1 h (PQQ-H_2_O_2_). Subsequently, the experimental groups were assessed for mitochondrial respiratory capacity using the Seahorse system, alongside measurements of ΔΨm. The resulting bioenergetic profiles demonstrated that PQQ pretreatment effectively mitigated the decline in mitochondrial respiratory capacity caused by oxidative stress. This protective effect was evidenced by a significant reduction in proton leak (71%), accompanied by an increase in ATP production (49%) and spare respiratory capacity (26%), though this latter parameter did not reach statistical significance (Figure 4A). These improvements suggest that PQQ may preserve the functional integrity of mitochondrial membranes and the electron transport chain (ETC), as the experimental medium mainly contains glucose and pyruvic acid, but not fatty acids.

Despite this positive modulation, significant reductions in ΔΨm were observed in both H_2_O_2_-treated samples (32% decrease vs. CTR) and samples pretreated with PQQ (30% decrease vs. CTR) (Figure 4B), indicating that similar ΔΨm values may correspond to distinct mitochondrial bioenergetic and functional states.

### 2.5. PQQ Pretreatment Upregulates the Protein Levels of SIRT3

Enhancing mitochondrial biogenesis is a fundamental strategy to support oxidative phosphorylation (OXPHOS) and ATP production by increasing the number of functional mitochondria. PQQ has been reported to promote mitochondrial biogenesis primarily through activation of the PGC-1α/SIRT1 signalling pathway [35]. Additionally, it can enhance mitochondrial function by upregulating SIRT3 expression, a key regulator of energy metabolism and mitochondrial homeostasis [40].

**Figure 5:**
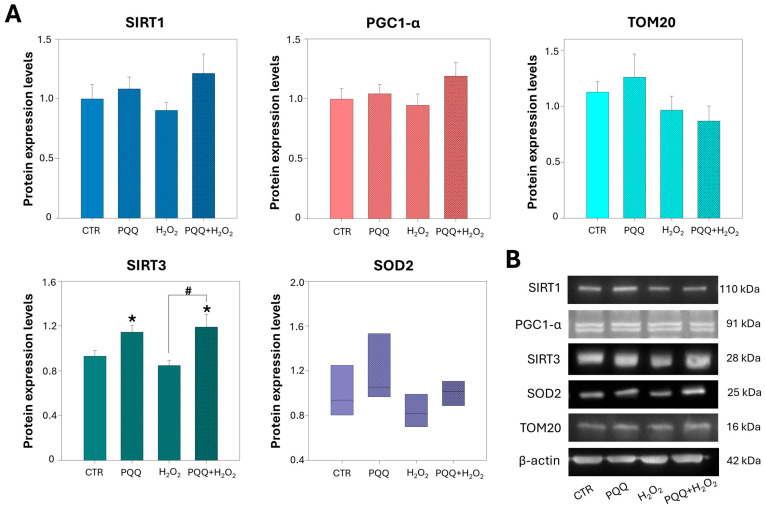
**PQQ pretreatment upregulates the expression of SIRT3 in HTM cells.** (**A**) Bar graphs represent protein levels of SIRT1, PGC1-α, TOM20, SIRT3, and SOD2, after 24 h of exposure to 100 nM PQQ, analysed by Western blotting. Both PQQ alone and PQQ + H_2_O_2_ co-treatment induced a significant increase in SIRT3 levels, whereas PGC1-α, SIRT1, TOM20, and SOD2 levels remained mostly unchanged. H_2_O_2_ exposure by itself did not affect any of these protein levels. Protein levels are expressed as relative units (r.u.). (**B**) Representative images of Western blot membranes; β-actin was used as an internal control. Data are shown as the mean ± SE from three independent experiments. One-way ANOVA followed by the Holm–Sidak post hoc test (* *p* < 0.05 vs. CTR; # *p* < 0.05). ANOVA on ranks performed for the analyses of SOD2 protein levels.

To elucidate the mechanisms by which PQQ enhances mitochondrial function after 24 h of exposure, we quantified the protein levels of PGC1-α, SIRT1, SIRT3, SOD2, and TOM20 (a marker of mitochondrial mass) by Western blotting. As shown in Figure 5A, both the PQQ and PQQ + H_2_O_2_ treatment groups exhibited a significant increase in SIRT3 expression and a slight modulation of SOD2 compared with controls, whereas PGC1-α, SIRT1, and TOM20 levels remained mostly unchanged. H_2_O_2_ treatment alone had no significant effect on the expression of any of these proteins. These findings suggest that the mitochondrial benefits of PQQ may be mediated through the SIRT3 signalling pathway and occur independently of classical mitochondrial biogenesis pathways.

### 2.6. PQQ Mitigates H_2_O_2_-Induced Morphological and Ultrastructural Damage to Mitochondria

The presence of cell subpopulations with reduced ΔΨm, along with distinct mitochondrial activity patterns and bioenergetic profiles, suggests the occurrence of underlying mitochondrial morphological alterations [44,45]. To test this hypothesis, we analysed mitochondrial ultrastructure, including cristae architecture, using transmission electron microscopy (TEM) and assessed mitochondrial network morphology via confocal microscopy.

**Figure 6:**
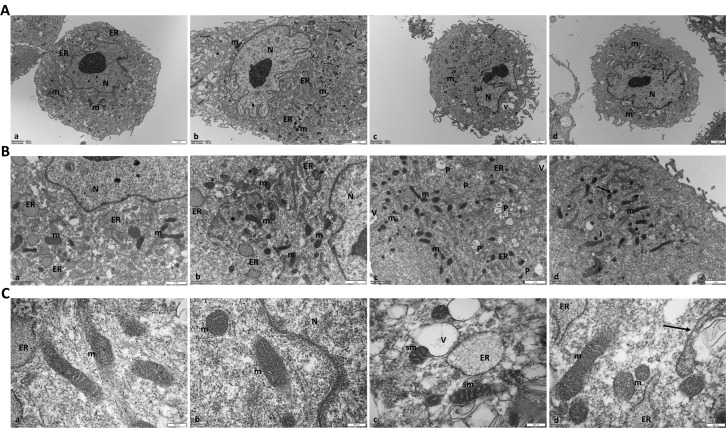
**PQQ alleviates H_2_O_2_-induced mitochondrial ultrastructural damage in HTM cells.** Panel (**A**): representative transmission electron microscopy (TEM) images (3400×) of control and treated HTM cells. (**a**) Control cells; (**b**) cells treated with 100 nM PQQ; (**c**) cells treated with 100 µM H_2_O_2_; (**d**) cells co-treated with 100 µM H_2_O_2_ and 100 nM PQQ. Scale bar = 2 µm. Panel (**B**): representative TEM images (10,500×) of mitochondrial morphology in control and treated cells. (**a**) In control cells, the mitochondria exhibit lamellar cristae with visible cristae membranes; (**b**) in cells treated with PQQ, the mitochondria are comparable to those in the control group. In some zones (*), the cristae are presumably cut tangentially and therefore not visualised by electron microscopy. (**c**) In cells treated with H_2_O_2_, the mitochondria seem to be smaller. The cristae of the membrane also appear thicker. Numerous vacuoles (V) and phagolysosomes (P) are present. (**d**) Combined treatment with PQQ and H_2_O_2_ appears to counteract the mitochondrial stress induced by H_2_O_2_ alone, leading to restoration of normal internal architecture (*) and causing some mitochondria to divide (arrow). Scale bar = 1 µm. Panel (**C**): TEM images showing details at higher magnification (46,000×) of the mitochondrial ultrastructure in control and treated cells. (**a**) Elongated mitochondria with normal internal architecture and visible lamellar cristae in control cells; (**b**) mitochondria in PQQ-treated cells similar to control mitochondria; (**c**) altered mitochondria with swollen, electron-dense cristae (sm) associated with vacuoles (V) in H_2_O_2_-treated cells; (**d**) restored (m) and dividing mitochondria (arrows) in PQQ + H_2_O_2_-treated cells. Scale bar = 200 nm. Abbreviations: N, nucleus; ER, rough endoplasmic reticulum; m, mitochondria; sm, swollen and electron-dense mitochondrial cristae; V, vacuoles; P, phagolysosomes.

Under TEM, control cells exhibit the ultrastructural features characteristic of metabolically active cells, including mitochondria with normal internal architecture and an abundance of rough endoplasmic reticulum (Figure 6A(a)). The latter appears as flattened cisternae and dilated vesicles dispersed throughout the cytoplasm. The cells also display a large eccentric nucleus with prominent chromatin, indented and irregular in its shape.

Similarly, PQQ-exposed groups exhibit the presence of numerous mitochondria and large quantities of endoplasmic reticulum vesicles (Figure 6A(b)). Conversely, treatment with H_2_O_2_ appears to alter the ultrastructure of the cell, affecting the nucleus, which becomes more indented and sometimes bilobed, and mitochondria, which appear smaller. Clear vacuoles are also observed in the cytoplasm (Figure 6A(c)). Finally, the combination of the two compounds seems to partially reverse the damage induced by H_2_O_2_ (Figure 6A(d)).

Figure 6B,C show the mitochondrial morphology of control and treated cells at higher magnification. While control cells exhibit normal internal mitochondrial architecture with visible lamellar cristae (Figure 6(B(a),C(a))), H_2_O_2_-treated cells exhibit smaller mitochondria with swollen cristae, which suggests possible mitochondrial dysfunction (Figure 6(B(c),C(c))). In addition to the altered mitochondrial ultrastructure, mitochondria associated with vacuoles (Figure 6C(c)) and numerous phagolysosomes are present in the cytoplasm (Figure 6B(c)).

Treatment of cells with PQQ alone does not seem to induce mitochondrial changes. PQQ pretreatment effectively mitigates the mitochondrial damage induced by H_2_O_2_, as evidenced by the restoration of normal mitochondrial size and shape and partial reorganisation of cristae architecture (Figure 6B(d),C(d)). In this context, dividing mitochondria are also observed, possibly suggesting active mitochondrial remodelling. Quantitative analysis supports the presence of altered mitochondria in H_2_O_2_-treated cells, showing a significant reduction in mitochondrial area, perimeter, and aspect ratio along with an increase in circularity (Table 1). Interestingly, PQQ pretreatment mitigated these changes, restoring all morphological parameters to levels comparable to control levels.

**Figure 7:**
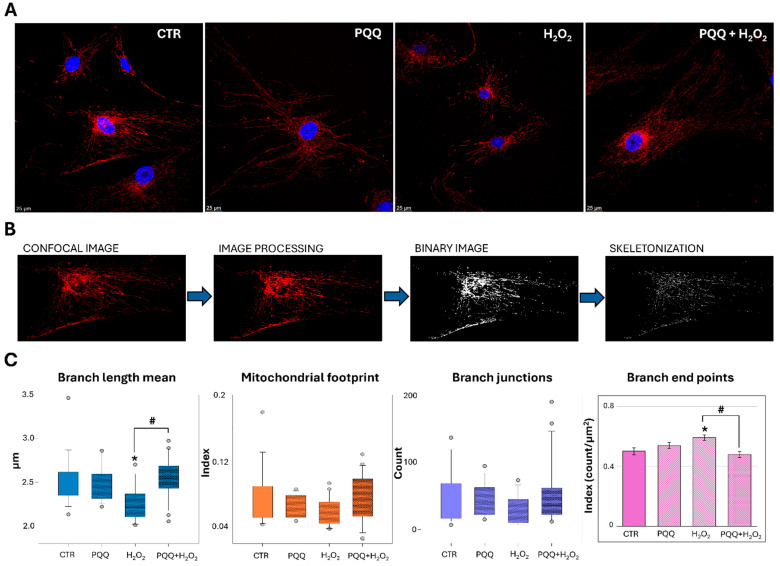
**PQQ mitigates hydrogen peroxide-induced morphological alterations of the mitochondrial network in HTM cells.** (**A**) Representative confocal microscopy images of the different experimental groups obtained by TOM20 (red) and DAPI (blue) staining. (**B**) Schematic representation of the computational skeletonisation workflow process performed using ImageJ (Fiji, version 1.51n, Bethesda, MD, USA). (**C**) Graphs show the quantification of “Branch Length Mean”, “Mitochondrial Footprint”, “Branch junctions”, and “Branch end points” parameters, analysed using MiNA and the Mitochondria Analyser plugin in ImageJ (Fiji, Bethesda, MD, USA). Data are presented as the mean of three independent experiments ± SE; statistical analysis was performed using ANOVA on ranks (Kruskal–Wallis test) followed by Dunn’s method (* *p* < 0.05 vs. CTR; # *p* < 0.05) or one-way ANOVA followed by the Holm–Sidak method (* *p* < 0.05 vs. CTR; # *p* < 0.05).

Analysis of mitochondrial network morphology further confirms that PQQ mitigates the structural alterations induced by H_2_O_2_ exposure. As shown in Figure 7A, both control and PQQ-treated cells display a predominantly tubular mitochondrial morphology, indicative of a healthy and interconnected network.

In contrast, mitochondria in H_2_O_2_-treated cells appear notably shorter and more fragmented, reflecting a significant disruption of network architecture. Quantitative analysis confirmed this observation, revealing a marked reduction in the Branch Length Mean alongside a significant increase in Branch end points (Figure 7C). No significant changes were observed in other parameters, including Mitochondrial Footprint and Branch junctions. Notably, PQQ pretreatment attenuated these alterations, restoring both Branch Length Mean and Branch end points towards control levels, thereby confirming the mito-protective effects previously attributed to PQQ.

### 2.7. Protective Effects of PQQ Against H_2_O_2_-Induced Cytotoxicity in HTM Cells

**Figure 8:**
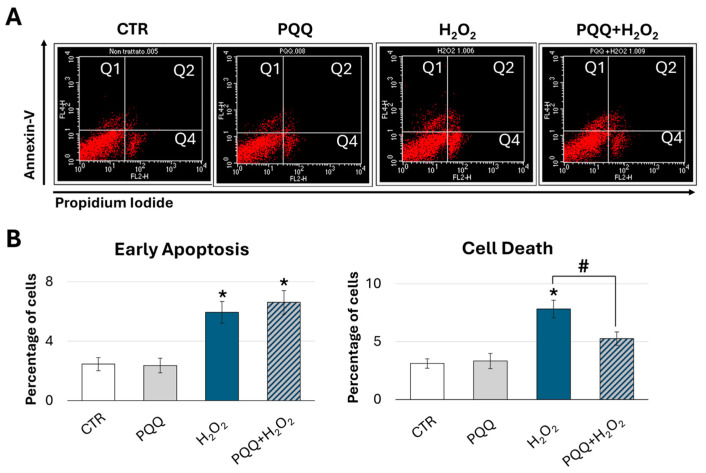
**Protective effects of PQQ against hydrogen peroxide-induced cytotoxicity in HTM cells.** (**A**) Representative flow cytometry images of HTM cells stained with Annexin V and Propidium Iodide, corresponding to the indicated experimental groups. Q1 = early apoptosis; Q2 + Q4 = Cell Death. (**B**) Bar graphs show the percentages of early apoptotic cells (Early Apoptosis) and late apoptotic/necrotic cells (Cell Death) in the different samples, assessed 24 h after H_2_O_2_ treatment. Data are expressed as the mean ± SE of three independent experiments. One-way ANOVA followed by the Holm–Sidak post hoc test (* *p* < 0.05 vs. CTR; # *p* < 0.05).

To further investigate the protective role of PQQ in promoting recovery from H_2_O_2_-induced damage, we evaluated levels of apoptosis and necrosis across the different experimental groups. At 24 h after treatments, cells were double stained with Annexin V and propidium iodide (PI) and analysed by flow cytometry (Figure 8A). Compared with the groups treated with H_2_O_2_ alone, PQQ pretreatment resulted in a significant 33% reduction in late apoptosis and necrosis (Q2 and Q4 quadrants) (Figure 8B). In contrast, no significant differences were observed in early apoptosis levels.

## 3. Discussion

Mitochondrial dysfunction and redox imbalance lead to bioenergetic collapse, driving the progressive decline in TM cell function, elevation in intraocular pressure, and, ultimately, irreversible vision loss. Among the tissues of the anterior chamber, the TM is particularly susceptible to oxidative damage, owing to its relatively limited antioxidant defence mechanisms [46,47,48]. Mitochondrial dysfunction and metabolic alterations in the TM are early pathological events in glaucoma development and play a critical role in driving the abnormal extracellular matrix (ECM) deposition observed in the onset of glucocorticoid-induced glaucoma [18,19,49,50]. Therefore, enhancing mitochondrial function and increasing cellular energy production represent a promising strategy to mitigate oxidative damage and preserve TM cell viability [51,52].

Our findings demonstrate that PQQ per se enhances mitochondrial respiratory capacity and increases ATP production in HTM cells. Furthermore, under oxidative conditions, PQQ confers significant cytoprotective effects against H_2_O_2_-induced damage by preserving mitochondrial function. That is evidenced by the normalisation of proton leak, increase in intracellular ATP levels and respiratory reserve capacity, partial amelioration of the mitochondrial ultrastructure, improved network morphology, and marked reduction in cell death. Although these protective effects are not associated with alterations in mitochondrial content or activation of the SIRT1–PGC-1α axis, they may be mediated through the upregulation of SIRT3, a critical regulator of mitochondrial bioenergetics and redox homeostasis. To the best of our knowledge, this study is the first to demonstrate the protective effects of PQQ against oxidative-stress-induced damage in HTM cells.

Overall, our findings align with previous studies that have highlighted the role of bioactive quinones, including PQQ, in modulating mitochondrial function, oxidative stress responses, and cellular senescence [24]. PQQ exerts antioxidant, neuroprotective, and cardiometabolic effects and has been shown to target genes involved in mitochondrial metabolism and fatty acid oxidation [53]. However, though PQQ cytoprotective properties at low (nanomolar) concentrations are well established, higher doses have been shown to elicit pro-apoptotic and antitumor activities [54,55]. Moreover, PQQ-mediated cytotoxicity is highly cell-type dependent; rat hippocampal neurons display negligible toxicity at concentrations up to 100 μM [56], whereas human SH-SY5Y neuroblastoma and rat PC-12 pheochromocytoma cells exhibit significant viability loss at ≥30 μM and ≥3 μM, respectively [57,58]. Consistent with these findings, exposure of primary mouse cortical neurons to 60 μM PQQ for 24 h also results in a marked reduction in cell viability [55]. Accordingly, the present study first explored the impact of a wide range of PQQ concentrations on selected indicators of cellular homeostasis, including cell viability, redox balance, and mitochondrial function.

Treatment with PQQ up to 10 μM did not affect cell proliferation or viability, indicating an absence of cytotoxicity. Furthermore, intracellular ROS levels remained unchanged under these conditions, suggesting that redox homeostasis was maintained. As previously described, mitochondrial metabolism has been identified as a primary target of PQQ. Given the presence of PQQ traces in human tissues, mitochondrial function was evaluated across a concentration range from 1 to 100 nM. HTM cells exposed to 100 nM PQQ exhibited significant increases in basal and maximal respiration, ATP-production-coupled respiration, and non-mitochondrial oxygen consumption, suggesting a positive remodelling of mitochondrial metabolic activity. Under these experimental conditions, a slight, though not significant, decrease in mitochondrial membrane polarisation was also observed compared with control samples. This phenomenon may reflect the dynamic nature of the mitochondrial proton circuit, where increased ATP production leads to a slight decrease in ΔΨm that is partially offset by enhanced electron transport and proton pumping, thereby maintaining ΔΨm at a near steady-state level. Indeed, while negative shifts in ΔΨm associated with the activation of oxidative phosphorylation are typically modest (approximately 10% reductions), oxygen consumption rates under these conditions often increase faster and much more substantially [43]. Overall, these results are consistent with previously published data demonstrating that PQQ can induce an ATP-boosting effect and enhance mitochondrial activity in various experimental models, both in vitro and in vivo [37,38].

However, the available literature on PQQ-mediated modulation of ΔΨm remains complex and somewhat difficult to interpret, as it describes biphasic effects. At low concentrations, PQQ generally supports or stabilises ΔΨm by enhancing mitochondrial function and promoting biogenesis [39]. In contrast, at higher concentrations, particularly in metabolically sensitive (i.e., primary cortical neurons) or tumoral cells, it may lead to a reduction in ΔΨm due to increased proton flux and ATP levels or even induce ΔΨm collapse as part of a cytotoxic response [37,59]. Regarding the observed increase in non-mitochondrial O_2_ consumption, it is known that the reduced form of PQQ (PQQH_2_) can be reoxidised by transferring electrons to molecular oxygen, leading to the formation of a superoxide anion, which is subsequently dismutated to hydrogen peroxide. This mechanism could account for the enhanced non-mitochondrial oxygen consumption observed.

Our findings also demonstrate that 100 nM PQQ confers a protective effect against oxidative stress-induced cell death in HTM cells, possibly through the restoration of mitochondrial function. H_2_O_2_ is one of the cytotoxic agents most widely used to induce cellular oxidative stress due to its high membrane permeability and its ability to react with free iron ions to generate highly reactive hydroxyl radicals. In TM cells, the concentrations of H_2_O_2_ used (100 µM to 1 mM) and exposure durations (0.5 to 24 h) vary widely among studies [47,60]. Regardless of these differences, exogenous oxidative stress consistently elicits a range of deleterious effects in TM cells, including reduced cellular metabolic activity, increased ECM synthesis, upregulation of inflammatory mediators, and reductions in mitochondrial function of up to 60% [47].

Under our experimental conditions, PQQ-mediated rescue of mitochondrial function after H_2_O_2_ exposure operates on two principal levels: restoration of respiratory capacity and enhancement of mitochondrial structure and network dynamics. Specifically, preservation of inner mitochondrial membrane integrity and electron transport chain function is evidenced by a 71% reduction in proton leak, accompanied by a 49% increase in ATP production and a 26% rise in spare respiratory capacity (SRC), though this latter parameter did not reach statistical significance. SRC, the difference between maximal and basal respiration, serves as a measure of a cell’s ability to meet elevated energy demands [61]. Because mitochondrial bioenergetics are highly dynamic, cells can rapidly mobilise SRC under acute stress to forestall ATP depletion. Thus, the PQQ-driven increase in SRC highlights an enhanced mitochondrial capability to supply additional energy beyond basal levels in response to oxidative challenge, thereby preventing an ATP crisis and subsequent cell-death events [61]. As a bioenergetic index of mitochondrial fitness, SRC depends on multiple parameters, notably the integrity of both the electron transport chain complexes and the inner mitochondrial membrane.

Unexpectedly, we observed comparable and significant reductions in ΔΨm in both H_2_O_2_-treated cells (−32% vs. CTR) and PQQ-pretreated cells (−30% vs. CTR) (Figure 4B). However, such equivalent ΔΨm decreases may mask distinct mitochondrial injury degrees and/or bioenergetic adaptations. Indeed, in cells exposed to H_2_O_2_ alone, ΔΨm loss coincides with a pronounced rise in proton leak and no enhancement in ATP production. Conversely, cells co-treated with PQQ and H_2_O_2_ exhibit ΔΨm depolarisation alongside significant increases in basal respiration and ATP synthesis, events indicative of improved electron transport chain flux and proton pump efficiency. This is further supported by a marked reduction in proton leak. Consistently, these functional enhancements align with parallel improvements in mitochondrial network morphology.

Mitochondrial morphology is continuously remodelled through fission and fusion events, which are essential for maintaining mitochondrial homeostasis and are tightly regulated in response to the cell’s bioenergetic and metabolic status [44,45,62]. While it is well established that a loss of ΔΨm can promote fragmentation by activating fission pathways, emerging evidence also suggests that mitochondrial dynamics can be driven by the activity of the FoF1–ATPase complex and independently of ΔΨm levels [45].

In this regard, confocal microscopy analysis of mitochondrial morphology revealed a predominantly fragmented phenotype in cells exposed to H_2_O_2_, accompanied by significant alterations in mitochondrial network architecture as also evidenced by decreased values in Branch Length Mean alongside a significant increase in Branch end points.

These effects were mitigated by PQQ pretreatment. In particular, PQQ-exposed cells displayed a reduced degree of fragmentation and preservation of mitochondrial network organisation. TEM analyses confirmed this trend, thus revealing that PQQ preserved the mitochondrial ultrastructure under oxidative stress. Control and PQQ-pretreated cells both maintained elongated organelles with intact outer membranes, densely packed cristae, and uniform electron-dense matrices, indicating retained bioenergetic integrity. In contrast, H_2_O_2_ exposure induced classic signs of mitochondrial injury, including organelle shortening, cristae disorganisation and dilation, matrix swelling, and peri-mitochondrial vacuolisation. Quantitative morphometric analysis further supported these findings, showing a significant reduction in mitochondrial area, perimeter, and aspect ratio, along with an increase in circularity in H_2_O_2_-treated cells consistent with a fragmented and rounded morphology. These morphological alterations may also reflect the activation of mitophagy, as suggested by the close association of damaged mitochondria with surrounding vacuoles. Notably, PQQ pretreatment normalised mitochondrial morphology, restored cristae architecture, and rescued the quantitative changes, suggesting that its protective effects involve the stabilisation of inner membrane structure and the maintenance of cristae integrity during oxidative challenge.

Upregulation of mitochondrial biogenesis is fundamental for optimising OXPHOS efficiency and elevating ATP synthesis. PQQ has been identified as a potent modulator of mitochondrial function, in part via activation of key biogenesis pathways such as the SIRT1–PGC1-α axis [34,35,37]. However, the effects of PQQ are highly context-dependent, varying with concentration, cell type, mitochondrial phenotypes, and cellular metabolic requests. In our trabecular meshwork model, a 24 h PQQ treatment failed to change mitochondrial content or the protein levels of PGC1-α and SIRT1. These findings are consistent with previous research indicating that PQQ does not effectively induce mitochondrial biogenesis at the transcriptional level, either in the short or long term, in retinal tissue [41]. Our data imply that PQQ enhances respiratory capacity and ATP output through mechanisms distinct from canonical biogenesis, most likely by improving electron transport chain efficiency and preserving membrane integrity under oxidative stress. In this respect, targeting SIRT3 may help preserve metabolic homeostasis in trabecular meshwork cells by restoring mitochondrial function. As a pivotal energy-sensing deacetylase, SIRT3 regulates mitochondrial function by deacetylating specific lysine residues on key respiratory proteins and acts as an activator of important proteins for the tricarboxylic acid cycle and fatty-acid oxidation. Notably, SIRT3 directly interacts with and deacetylates NADH dehydrogenase subunit A9 (NDUFA9), enhancing complex I activity, and cytochrome c oxidase subunit 4 isoform 2 (COX4I2), thereby promoting mitochondrial respiration and sustaining basal ATP production [63]. Moreover, SIRT3 enhances mitochondrial antioxidant defence by deacetylating and activating superoxide dismutase 2 (SOD2), thereby promoting the detoxification of reactive oxygen species [63]. PQQ-exposed groups exhibit an increase in SIRT3 protein expression, suggesting its possible involvement in mediating the enhancing effects of mitochondrial fitness induced by PQQ. These results are in line with a recent work by Wang and colleagues showing that PPQ achieves protective effects in HK-2 cells through the PI3K/Akt/FoxO3a pathway and SIRT3-dependent modulation [40]. In our experimental model, however, further investigation of SIRT3 enzymatic activity, along with the use of pharmacological SIRT3 inhibitors, is necessary to confirm its specific involvement. Furthermore, the activation of alternative signalling pathways, including PI3K/Akt/FoxO3a, may be helpful to deeply understand the adaptive mitochondrial response of PQQ.

## 4. Materials and Methods

### 4.1. Cell Culture and Treatments

The primary human trabecular meshwork (HTM) cell line (P10879) and its specific culture medium kit (P60108) were obtained from Innoprot (Derio, Bizkaia, Spain). For routine culture, the medium was supplemented with 2% foetal bovine serum (FBS), 1% Fibroblast Growth Supplement (FGS), and 1% antibiotic solution (penicillin 10,000 U/mL, streptomycin 10,000 μg/mL) purchased from Innoprot (Derio, Bizkaia, Spain). Cells were maintained at 37 °C in a humidified atmosphere containing 5% CO_2_ and cultured until reaching a minimum confluence of 80%, with medium changes every 2–3 days [64]. All experiments were conducted using HTM cells up to passage 15, in accordance with the supplier’s recommendations. To improve cell adhesion, culture surfaces were pre-coated with poly-L-lysine (Innoprot, Derio, Bizkaia, Spain), following the manufacturer’s instructions. Cell viability and proliferation were routinely assessed using the Trypan Blue exclusion test and the MTT (3-(4,5-dimethylthiazol-2-yl)-2,5-diphenyl-2H-tetrazolium-bromide) assay. For experimental procedures, HTM cells were seeded and incubated in complete culture medium for 24 h before treatments. Cells were then exposed to a range of PQQ (Merck KGaA, Darmstadt, Germany) concentrations (from 1 nM to 10 µM) for 24 h to evaluate the effects on proliferation, viability, and ROS generation. To induce oxidative stress, HTM cells were treated with a broad range of H_2_O_2_ (Sigma-Aldrich, Darmstadt, Germany) concentrations, from 100 µM to 1600 µM, for 1 h. After an additional 24 h incubation, cell proliferation and cytotoxicity were assessed to generate a dose–response curve. For all subsequent experiments, cells were pretreated with 100 nM PQQ for 23 h, followed by co-exposure to 100 µM H_2_O_2_ for 1 h.

### 4.2. Detection of Intracellular Reactive Oxygen Species (ROS)

Intracellular reactive oxygen species (ROS) generation was evaluated using 2′,7′-dichlorofluorescin diacetate (DCFH-DA; ThermoFisher Scientific, Waltham, MA, USA), as previously described [11,12]. Briefly, immediately following the experimental treatments, cells were incubated with 5 μM DCFH-DA at 37 °C for 30 min in the dark. After incubation, cells were collected and washed twice with cold PBS (pH 7.4) and resuspended in PBS. Fluorescence intensity was measured using a spectrofluorometer (Perkin-Elmer LS-50B; Waltham, MA, USA) with excitation and emission wavelengths set at 502 nm and 524 nm, respectively, to quantify ROS levels.

### 4.3. Mito Stress Test by Seahorse XF96 Extracellular Flux Analyser

For bioenergetic profiling, HTM cells were seeded into poly-L-lysine-pretreated XF 96-well cell culture microplates (Agilent Seahorse, Santa Clara, CA, USA). The Mito Stress Test was performed using an XF96 Extracellular Flux Analyser (Agilent Seahorse), and oxygen consumption rate (OCR) was measured in XF Assay Medium (#102365, Agilent Seahorse) containing 1 mM pyruvate, 2 mM glutamine, and 10 mM glucose. OCR values (expressed as pmol/min/10^4^ cells) were recorded following the sequential injection of the following mitochondrial inhibitors: 1.5 μM oligomycin A (#75351, Sigma-Aldrich, St. Louis, MO, USA), 75 μM 2,4-dinitrophenol (2,4-DNP; #D198501, Sigma-Aldrich, St. Louis, MO, USA), 0.5 μM rotenone (#R8875, Sigma-Aldrich, St. Louis, MO, USA), and 0.5 μM antimycin A (#A8674, Sigma-Aldrich, St. Louis, MO, USA). Basal respiration was calculated as the last OCR measurement obtained prior to the injection of oligomycin A, after subtraction of non-mitochondrial respiration. ATP production was derived by subtracting the minimum OCR value following oligomycin A administration from the last OCR value measured before the injection. Proton leak was calculated as the minimum OCR after oligomycin A injection, again corrected by subtracting the non-mitochondrial component. All OCR values were normalised to the number of cells.

### 4.4. Flow Cytometry Analyses

All cytofluorimeter experiments were performed using a FACSCalibur flow cytometer (Becton Dickinson Instruments Inc., Franklin Lakes, NJ, USA). Data related to cell death analysis were processed using ModFit LT for Mac (version 3.0), whereas mitochondrial membrane potential (ΔΨm) measurements were acquired with CellQuest software (version 3.3) (BD Instruments Inc., Franklin Lakes, NJ, USA).

Forward and side scatter channels were gated to include the main population of cells with normal size and granularity. For each experimental condition, a minimum of 10,000 events per sample were collected and analysed.

#### 4.4.1. Assessment of Mitochondrial Membrane Potential (ΔΨm) by Flow Cytometry

ΔΨm was assessed using the JC-1 fluorescent probe (ThermoFisher Scientific, Waltham, MA, USA). Both treated and control cells were incubated with 3 μM JC-1 for 30 min at 37 °C in a humidified atmosphere. Following incubation, cells were trypsinised, washed with PBS, and immediately analysed by flow cytometry. JC-1 monomer fluorescence (green) and aggregate fluorescence (red) were detected using the FL-1 (bandpass filter 525/65 nm) and FL-2 (bandpass filter 575/65 nm) channels, respectively. We used 10 μM carbonyl cyanide m-chlorophenyl hydrazone (CCCP) (Sigma-Aldrich, St. Louis, MO, USA) for 1 h as a positive control to induce ΔΨm dissipation. Data are expressed as the mean red/green fluorescence ratio, calculated as the ratio of the mean fluorescence intensity (MFI) in the red and green channels, respectively.

#### 4.4.2. Determination of Cell Death by Annexin V/Propidium Iodide Assay

At 24 h after PQQ exposure to H_2_O_2_ alone and the combined treatment, cell death was assessed using the eBioscience™ Annexin V Apoptosis Detection Kit APC (Thermo Fisher Scientific, Waltham, MA, USA). Cells were detached by trypsinisation, washed with PBS, and resuspended in the supplied binding buffer. Each sample (1 × 10^6^ cells) was incubated with Annexin V-APC and Propidium Iodide for 15 min at room temperature in the dark, as described by the manufacturer’s instructions. Following incubation and subsequent washing, samples were analysed by flow cytometry. As a positive control for apoptosis induction, cells were exposed to 50 μM etoposide for 24 h.

### 4.5. Western Blot Analysis

Protein concentration was determined by the BCA protein assay kit (Pierce, Rockford, IL, USA). Equal amounts of protein were separated by SDS-PAGE and transferred to polyvinylidene difluoride (PVDF) membranes (Sigma-Aldrich, St. Louis, MO, USA). Non-specific binding sites were blocked for 1 h at room temperature with 5% non-fat dry milk (Bio-Rad Laboratories, Segrate, Italy) in Tris-buffered saline containing 0.05% Tween 20 (TBS-T). Membranes were incubated with polyclonal rabbit anti-SIRT1 antibody (Ab189494, Abcam, Cambridge, UK; 1:1000), anti-SIRT3 antibody (ARC51535, Avantar; 1:1000), anti-PGC1-α antibody (A87835, Antibodies, 1:1000), anti-TOM20 antibody (PA5-52843, ThermoFisher Scientific, Waltham, MA, USA; 1:1000), and anti-β-Actin antibody (Ab8227, Abcam, Cambridge, UK; 1:3000) overnight at 4 °C, followed by incubation with horseradish peroxidase (HRP)-conjugated anti-rabbit (BA1054, Boster Biological Technology Co., Ltd., Pleasanton, CA, USA, 1:2000) for 1 h at room temperature. After washing, specific immunoreactive complexes were detected using an ECL kit (Thermo Scientific, Waltham, MA, USA) and a Uvitec Cambridge system (Alliance series, Cambridge, UK). The bands were normalised for β-Actin using ImageJ 1.44 p software, and values are given as relative units (r.u.). All the experiments were performed in triplicate.

### 4.6. Transmission Electron Microscopy (TEM)

HTM cells were processed for transmission electron microscopy (TEM) analysis according to Ragusa et al. (2025) [65]. Briefly, control and treated cells were detached from plates and fixed in suspension with 2.5% glutaraldehyde (Electron Microscopy Sciences, EMS, Hatfield, PA, USA) in PBS for at least 48 h at 4 °C. After several washes in PBS, cells were post-fixed with 2% osmium tetroxide (EMS) for 2 h, dehydrated through an ascending series of alcohol washes, immersed in propylene oxide (Sigma-Aldrich, St. Louis, MO, USA) for 40 min, and left overnight in a 1:1 solution of propylene oxide/epoxy resin (Agar Scientific, Stansted, UK) (first resin). The first resin was removed, and samples were embedded in epoxy resin (Agar Scientific, Stansted, UK) alone for 48 h at 60 °C. Resin blocks were cut with a diamond knife in ultrathin sections (90–100 nm) using an Ultracut E ultramicrotome (Leica EMUC6, Wetzlar, Germany). Ultrathin sections were mounted on 100-mesh copper grids (Assing, Rome, Italy), contrasted using Uranyless (a uranyl acetate alternative) (TAAB Laboratories Equipment Ltd., Aldermaston, UK) and lead citrate (Electron Microscopy Sciences, Hatfield, PA, USA), and analysed using a TEM (Philips CM100, Philips Electron Optics B.V., Eindhoven, The Netherlands)) operating at 100 kV. Images were acquired using a PHURONA digital camera (Emsis) equipped with a CMOS sensor (3008 × 4112 pixels, maximum resolution) and a frame rate ranging from 20 fps (at full resolution) to 50 fps (in binned mode). Mitochondrial shape descriptors and size measurements were obtained using Image J software as previously reported [66,67] by manually tracing mitochondria from TEM images. Surface area (mitochondrial size) is reported in squared micrometres, perimeter is reported in micrometres, aspect ratio (AR) was computed as [(major axis)/(minor axis)] and reflects the ‘length-to-width ratio’, and circularity [4π·(surface area/perimeter^2^)] is an index of sphericity, with a value of 1 indicating a perfect spheroid.

### 4.7. Immunofluorescence

HTM cells were seeded onto poly-L-lysine-coated glass coverslips and subjected to the indicated treatments. After incubation, cells were fixed with 4% paraformaldehyde (PFA) in PBS for 10 min at room temperature (RT), followed by permeabilisation with 0.1% Triton X-100 in PBS for 5 min at RT. Non-specific binding sites were blocked using 10% bovine serum albumin (BSA) in PBS for 10 min at RT. Cells were then incubated overnight at 4 °C with a rabbit polyclonal anti-TOM20 primary antibody (1:500; Thermo Fisher Scientific, Waltham, MA, USA; PA5-52843). After washing, cells were incubated in the dark for 30 min at RT with a goat anti-rabbit Alexa Fluor 633-conjugated secondary antibody (1:2000) (Immunological Sciences, Rome, Italy). Nuclei were stained using Vectashield Mounting Medium with DAPI (Vector Laboratories, Inc., 30 Ingold Road, Burlingame, CA, USA). Confocal images were acquired using a Leica Stellaris 8 microscope (Leica Microsystems, Wetzlar, Germany) with a White Light Laser (WLL) and processed with LAS X software (version 4.7.0.28176). Mitochondrial morphology was analysed using a semi-automated Fiji workflow as previously reported [68,69,70], combining the Mitochondria Analyser (v2.1.0) and MiNA (StuartLab; ImageJ version 1.51n) plugins. Preprocessing included Unsharp Mask, CLAHE, and Median Filter, followed by binarisation (default threshold) to extract mitochondrial mass, indicated as the Mitochondrial Footprint. The resulting binary images were then skeletonised. Subsequently, quantitative parameters, including Branch Length Mean, Branch junctions, and Branch end points, were analysed. According to the Mitochondria Analyser user manual, the parameter Branch junctions is defined as “number junctions within mitochondrion’s skeleton where two or more branches meet”. A Branch end point is the “total number of end-point in mitochondrion’s skeleton where branches end without connecting to another branch”. The Mitochondrial Footprint was calculated as the index of mitochondrial mass (µm^2^) normalised on total cell area (µm^2^). Branch end points were calculated as the index between the number of branch endpoints normalised on mitochondrial total area (µm^2^).

### 4.8. Statistical Analysis

All statistical analyses were based on at least three independent experiments, each with a minimum of three technical replicates, except for Seahorse analyses, which included at least five technical replicates per condition. Unless otherwise stated, data are presented as the mean ± standard error of the mean (SEM). Statistical comparisons between group means were carried out using SigmaStat version 2.03 (SPSS Inc., Chicago, IL, USA). Student’s *t*-test and multiple paired *t*-tests were used to analyse statistical significance between two groups. For comparisons involving more than two groups, one-way ANOVA followed by the Holm–Sidak post hoc test or ANOVA on ranks (the Kruskal–Wallis test) followed by Dunn’s method was applied. Hydrogen peroxide dose–response curves and inhibitory concentration (IC) values on cell viability were obtained using OriginPro software, version 8.5 (OriginLab Corporation, Northampton, MA, USA). A *p*-value < 0.05 was considered statistically significant.

## 5. Conclusions

In summary, our study confirms mitochondria as direct targets of PQQ and reveals cytoprotective effects against oxidative stress-induced damage in HTM cells. Moreover, this work provides evidence for the possible involvement of SIRT3 signalling in restoring mitochondrial homeostasis and promoting an adaptive response to oxidative challenge. Taken together, these data support the potential of PQQ to represent a promising candidate for further investigation in in vivo animal models. Moreover, further studies are essential to determine the optimal dosing and delivery strategies in humans to replicate the mitochondrial and ATP-enhancing effects observed in vitro, both in physiological and pathological contexts, before PQQ can be considered a viable candidate for clinical application.

## Figures and Tables

**Table 1:** **Quantitative analysis of mitochondrial morphology in HTM cells.**

	CTR	PQQ	H_2_O_2_	PQQ + H_2_O_2_
**Area (µm^2^)**	0.081 [0.054–0.110]	0.088 [0.060–0.125]	0.042 * [0.030–0.066]	0.072 ^#^ [0.044–0.115]
**Perimeter (µm)**	1.125 [0.913–1.449]	1.212 [0.954–1.487]	0.786 * [0.665–1.006]	1.081 ^#^ [0.789–1.491]
**Circularity**	0.801 [0.657–0.855]	0.785 [0.652–0.855]	0.855 * [0.798–0.888]	0.800 ^#^ [0.647–0.871]
**Aspect ratio**	1.733 [1.416–2.390]	1.798 [1.430–2.583]	1.392 * [1.194–1.765]	1.678 ^#^ [1.321–2.528]

Quantitative assessment of mitochondrial morphology in HTM cells following exposure to H_2_O_2_, with or without PQQ treatment, was carried out. Mitochondrial shape and size parameters were measured using ImageJ software and include surface area (mitochondrial size), aspect ratio (AR, indicating elongation), circularity (values closer to 1 represent more spherical shapes), and perimeter (reflecting mitochondrial boundary complexity). Data are presented as the median (25th–75th percentile) from at least three independent experiments (for each condition, a minimum of 200 mitochondria were evaluated). Experimental groups were compared using Kruskal–Wallis ANOVA on ranks, with Dunn’s post hoc tests where appropriate (* *p* < 0.05 vs. CTR; # *p* < 0.05 vs. H_2_O_2_).

## Data Availability

All data produced are reported as results in this paper.
